# “You should go so that others can come”; the role of facilities in determining an early departure after childbirth in Morogoro Region, Tanzania

**DOI:** 10.1186/s12884-015-0763-1

**Published:** 2015-12-09

**Authors:** Shannon A. McMahon, Diwakar Mohan, Amnesty E. LeFevre, Idda Mosha, Rose Mpembeni, Rachel P. Chase, Abdullah H. Baqui, Peter J. Winch

**Affiliations:** Department of International Health, Johns Hopkins Bloomberg School of Public Health, 615 N. Wolfe Street, Baltimore, MD 21205-2179 USA; School of Public Health and Social Sciences, Department of Behavioural Sciences, Muhimbili University of Health and Allied Sciences, P.O. Box 65015, Dar-Es-Salaam, Tanzania; School of Public Health and Social Sciences, Department of Epidemiology and Biostatistics, Muhimbili University of Health and Allied Sciences, P.O. Box 65015, Dar-Es-Salaam, Tanzania; International Center for Maternal and Newborn Health, Department of International Health, Johns Hopkins Bloomberg School of Public Health, 615 N. Wolfe Street, Baltimore, MD 21205-2179 USA; Institute of Public Health, Ruprecht-Karls-Universität, Heidelberg, Germany

**Keywords:** Early discharge, Tanzania, Maternal health, Neonatal health, Length of stay

## Abstract

**Background:**

Tanzania is among ten countries that account for a majority of the world’s newborn deaths. However, data on time-to-discharge after facility delivery, receipt of postpartum messaging by time to discharge and women’s experiences in the time preceding discharge from a facility after childbirth are limited.

**Methods:**

Household survey of 1267 women who delivered in the preceding 2–14 months; in-depth interviews with 24 women, 12 husbands, and 5 community elders.

**Results:**

Two-thirds of women with vaginal, uncomplicated births departed within 12 h; 90 % within 24 h, and 95 % within 48 h. Median departure times varied significantly across facilities (hospital: 23 h, health center: 10 h, dispensary: 7 h, *p* < 0.001).

Quantitative and qualitative data highlight the importance of type of facility and facility amenities in determining time-to-discharge. In multiple logistic regression, level of facility (hospital, health center, dispensary) was the only significant predictor of early discharge (*p* = 0.001). However across all types of facilities a majority of women depart before 24 h ranging from hospitals (54 %) to health centers (64 %) to dispensaries (74 %). Most women who experienced a delivery complication (56 %), gave birth by caesarean section (90 %), or gave birth to a pre-term baby (70 %) stayed longer than 24 h. Reasons for early discharge include: facility practices including discharge routines and working hours and facility-based discomforts for women and those who accompany them to facilities. Provision of postpartum counseling was inadequate regardless of time to discharge and regardless of type of facility where delivery occurred.

**Conclusion:**

Our quantitative and qualitative findings indicate that the level of facility care and comforts existing or lacking in a facility have the greatest effect on time to discharge. This suggests that individual or interpersonal characteristics play a limited role in deciding whether a woman would stay for shorter or longer periods. Implementation of a policy of longer stay must incorporate enhanced postpartum counseling and should be sensitive to women’s perceptions that it is safe and beneficial to leave hospitals soon after birth.

**Electronic supplementary material:**

The online version of this article (doi:10.1186/s12884-015-0763-1) contains supplementary material, which is available to authorized users.

## Background

Time-to-discharge after childbirth in a health facility has been debated in medical journals and popular media for more than 50 years [1]. Perceptions of an “early” discharge vary across countries (and studies) and can range from 12 h up to 4 days [2]. While consensus has been reached on objectives of a postpartum stay – to monitor maternal and newborn health, and to provide counseling on breastfeeding and danger signs – no global standards have been reached on a number of hours or days sufficient to complete these objectives [3]. A technical consultation by the World Health Organization (WHO) recommended that for an uncomplicated delivery of a healthy, term baby, mother-baby pairs should stay under observation for 24 to 48 h; in cases of an earlier departure, a qualified professional should assess the dyad within 2 days of discharge [3].

Two systematic reviews, drawing largely on data from high-income settings, have failed to demonstrate a link between early discharge and maternal or neonatal morbidity or mortality [4, 5]. However, a Cochrane review, updated in 2008, found that if early discharge was paired with a home visit, there was no adverse impact on breastfeeding or maternal depression [2]. In low resource settings, early discharge data is limited. In searches of PubMed and Scopus, we found no studies on time to discharge from low-income countries.

More than 98 % of all maternal and newborn deaths occur in low-income settings [6, 7]; nearly half in sub-Saharan Africa. On the first day of life - described as “the most dangerous day for mothers and babies” [8] - an estimated 45–50 % of maternal deaths [9, 10] and 24–45 % of neonatal deaths [11–13] occur. A study in Bangladesh found that maternal mortality was “more than 100 times higher on the first day of birth and 30 times higher on the second day after birth than in the second year postpartum” [10]. Given that “the magnitude of mortality in the first and second day following birth is staggering” [14], studies have underlined that skilled attendance is valuable not only at delivery but also in the immediate postpartum period (for at least 24 h after birth) and that discharge before this time presents a missed opportunity to monitor mother-baby pairs, and provide valuable counseling and support to families [8].

Tanzania is the fifth most populous country in Sub–Saharan Africa, and is among 10 countries that account for a majority of the world’s newborn deaths [8]. For every 1,000 live births, 68 deaths occur before age 5; 40 % within the first 28 days and 14 % within the first 24 h of life [8]. One in 38 Tanzanian women die due to complications in pregnancy or childbirth (the “lifetime risk of maternal death”) [8], and for every 100,000 live births, 454 maternal deaths occur [15]. While receipt of at least one antenatal care session is nearly universal (96 %), only half of deliveries occur in health facilities [15]. There is a paucity of data on time to discharge. While the country’s National Postnatal Care Guidelines do not explicitly require a duration of stay, emphasis is placed on “assessment and close monitoring” of mother-baby pairs for 24 h [16].

This research seeks to provide evidence and recommendations to policy makers, practitioners and program staff as they craft policies and design interventions relevant to the immediate post-delivery period in Tanzania and similar settings. The more immediate aim is to fill a gap in the literature by presenting findings on time to discharge, characteristics of women who depart early, receipt of postpartum messaging and the experience of post-delivery facility departure as described by women in rural Tanzania.

## Methods

### Setting

This research was conducted in four districts of rural Morogoro Region, Tanzania: Morogoro Rural, Mvomero, Kilosa and Ulanga districts. These districts were chosen based on their inclusion in an integrated maternal and newborn health care program implemented by the Ministry of Health and Social Welfare (MoHSW) and MAISHA through Jhpiego (and which this research seeks to inform). In the region, nearly one quarter of women have no formal education, about a third (34 %) have incomplete primary education, another third (33 %) have completed primary school, 8 % have some secondary education and less than 1 % have completed secondary school [15]. While a majority of women can read a whole sentence, 26 % of women cannot read [15]. More than 60 % of men and women are engaged in agriculture as their primary occupation [15]. First- level health facilities in Tanzania are dispensaries, followed in ascending order by health centers, district and regional hospitals. Nationwide, cesarean sections are performed in most hospitals (92 %), few health centers (13 %) and no dispensaries [17]. More than half of hospitals have needles and syringes, intravenous solutions, injectable oxytocics, anticonvulsants and oral or injectable antibiotics in the delivery room compared with less than 20 % of health centers and less than 10 % of dispensaries [17]. Blood transfusion services are available at a majority of hospitals (99 %), few health centers (12 %) and nearly no dispensaries (1 %) [17].

### Sampling

A household survey was administered to 1,968 recently-delivered women (RDW) - women who gave birth, irrespective of outcome, within the past 14 months but not within the previous two months - across four districts. These women represent roughly 4 % of all pregnant women in the target districts. The survey was designed as a multistage cluster sampling survey, with the intention to collect baseline data on health indicators for a larger maternal health evaluation. Sixty clusters were selected through probability proportional to size (PPS) sampling methods and in each cluster, 30–35 RDWs were interviewed from August 2011 to November 2011. The survey team visited all households in each selected cluster to identify eligible women. If a household had more than one eligible woman, the interviewer compiled a list of the eligible women in the household and randomly selected one from the list. The survey included questions related to place and type of delivery, receipt of care throughout the maternal health continuum, duration of stay after delivery and several questions relevant to socioeconomic status and demographic characteristics (see Additional file 1. Quantitative Tool (Excerpts)).

The qualitative study sample included in-depth interviews with women who delivered in a facility in the past 14 months (*n* = 24), their husbands (*n* = 12) and community leaders (*n* = 5) across four districts in areas receiving additional health system inputs from an NGO and comparison arms. Qualitative data collection took place in July and August 2011. Researchers engaged with village health committees to identify eligible participants. Using open-ended guides, interviewers focused on capturing narratives related to the careseeking experience during antenatal, delivery and postnatal periods. Interviews were conducted at a time and place of the respondent's choosing, and typically lasted 90 min. Qualitative methods were used to understand how women described their discharge and the decision surrounding time to discharge with questions such as “Please describe the time period from the moment the baby was born until the time you left the facility” and “Please describe factors that made you want to stay in the facility or leave the facility after giving birth.” Lines of questioning were iterative and included probes such as “Please tell me more about that” (see Additional file 2. Qualitative Tool). Data collectors were fluent in Swahili, conversant in English and possessed university-level training in fields including: education, public health, and social sciences. Data collectors obtained informed written consent from respondents before conducting interviews or surveys.

### Analysis

For quantitative analysis, data from 1267 women who delivered at facilities were included. For all variables of interest, proportions and confidence intervals were calculated using the survey commands of Stata 12, which adjusted for clustering [18]. When calculating differences in median time to departure by type of facility, we used the Kruskal-Wallis test as data were right-skewed. Statistical significance was tested using bivariate logistic regression with p-values of less than 0.05 taken to be significant and incorporated into a multiple logistic regression model. In choosing a parsimonious model, we sought to include variables significant at alpha of 0.05, in either bivariate logistic analysis or in a full multiple logistic regression that included all variables of interest. We also conducted forward selection and backwards elimination stepwise regression using a cutoff value of *p* = 0.10 in both directions to further inform our final model selection. Using a chi-squared test, we assessed whether women received more postpartum care messages by staying the recommended 24 h or more. We restricted our multivariate analysis to women who experienced non-complicated vaginal deliveries. Women who delivered via cesarean section, vacuum extraction, reported a delivery complication or experienced a pre-term birth were analyzed separately for duration of stay due to sample size limitations and inherent differences across the two populations.

For qualitative analysis, a field supervisor led daily debriefing sessions with interviewers to triangulate findings, improve lines of inquiry for future interviews, build field notes and inform early drafts of a codebook. All qualitative interviews were recorded, transcribed, translated and coded using Atlas.ti [19]. Codes were applied and checked by a qualitative supervisor. Qualitative analysis was informed by the five stages of the framework approach: familiarization, theme identification, indexing, charting and interpretation [20]. A theoretical perspective guiding all analysis was the Social Ecological Model, which emphasizes levels of influence on behavior (individual, dyadic, environmental and structural) [21].

The study received ethical approval from the Muhimbili University of Health and Allied Sciences and Johns Hopkins School of Public Health Institutional Review Boards.

## Results

### Household survey: Facility type as a stronger predictor of early discharge than patient characteristics

Among women who delivered in a facility (*n* = 1267), 1152 (90.9 %) experienced normal, non-complicated vaginal births, 102 (8 %) had caesarean sections, and 5 (0.4 %) had forceps/suction assisted deliveries. Unlike women who did not experience a complication during delivery, a majority of women (64.5 %) who underwent a Cesarean section or suffered a delivery complication while giving birth in a facility were discharged after 24 h (see Table 1). Among these women, 120 (45 %) delivered in a hospital, 82 (31 %) in a health center and 65 (24 %) in a dispensary. While the study did not have enough power to examine factors that contribute to length of stay, even with a small sample the effect of higher level of facility care was strong.

**Table 1:** Post delivery stay by delivery characteristics

		Stayed < 24 hour n (row %)	Stayed ≥ 24 hours n (row %)	*P* value
Delivery Type	Normal vaginal delivery	692 (62.7)	413 (37.3)	<0.001
	Cesarean section	10 (10.5)	85 (89.5)	
	Assisted vaginal delivery	2 (40.0)	3 (60.0)	
Delivery Complications^a^	No	620 (61.2)	393 (38.8)	<0.001
	Yes	83 (44.1)	105 (55.9)	
Birth Timing	Preterm birth	9 (30.0)	21 (70.0)	0.001
	Term	700 (59.5)	477 (40.5)	

^a^severe vaginal bleeding, eclampsia, obstructed labor, retention of placenta, severe anemia and loss of consciousness

Among women who experienced a vaginal, non-complicated delivery in a health facility, a majority (65.7 %) were discharged within 24 h (See Table 2). Two thirds of these women departed within 12 h, 90 % within 24 h, and 95 % within 48 h. Median departure times varied across facilities and were significantly different (hospital: 23 h, health center: 10 h, dispensary: 7 h, Kruskal-Wallis test *p* < 0.001). In bivariate analysis, variables found to be significantly associated with a longer stay (*p* < 0.05) included delivering at a higher- level health facility (longer stay at hospital vs. health center, and at health center vs. dispensary), younger maternal age, any maternal education and lower parity. In the full multiple logistic regression model, delivery at a higher-level facility and younger maternal age remained significant. Based on these results, we chose to retain level of facility, maternal age, maternal education, and parity in our final model. Both forward selection and backward elimination stepwise regression agreed on a slightly more parsimonious model nested within our own that omitted parity as a predictor.

**Table 2:** Post delivery discharge^a^ Factors associated with stays of greater than 24 h among women who experienced a normal, full-term delivery^b^and had no complications during birth (*N* = 907). Only predictors that were significant (*p* < 0.05) in either bivariate analysis or the full model^c^ were included in the final model

	Stayed < 24 hours Frequency (%)	Stayed ≥ 24 hours Frequency (%)	Crude Odds Ratio^d^ (95 % CI), bivariate logistic regression	Adjusted Odds Ratio (95 % CI), final multiple logistic regression model
	596 (65.7)	311 (34.3)		
Place of delivery				
			*p < 0.001*	*p < 0.001*
Hospital	124 (53.9)	106 (46.1)	1	1
Health Center	186 (63.7)	106 (36.3)	0.67 (0.42–1.05)	0.70 (0.45–1.10)
Dispensary	286 (74.3)	99 (25.7)	0.40 (0.27–0.61)	0.43 (0.29–0.65)
Education^e^				
			*p = 0.013*	*p = 0.070*
None	153 (73.2)	56 (26.8)	1	1
Some Primary	58 (63.7)	33 (36.3)	1.55 (0.95–2.55)	1.40 (0.84–2.33)
Primary Complete	344 (63.1)	201 (36.9)	1.60 (1.21–2.11)	1.47 (1.11–1.96)
Secondary or Higher	32 (65.3)	17 (34.7)	1.45 (0.84–2.51)	1.26 (0.72–2.19)
Age				
			*p = 0.015*	*p = 0.140*
≤19	74 (54.8)	61 (45.2)	1	1
20–33	414 (67.1)	203 (32.9)	0.59 (0.42–0.85)	0.61 (0.38–0.99)
34–49	107 (69.5)	47 (30.5)	0.53 (0.32–0.88)	0.64 (0.33–1.23)
Parity				
			*p = 0.064*	*p = 0.154*
1	123 (61.2)	78 (38.8)	1	1
2–3	236 (63.1) 2	138 (36.9)	0.92 (0.66–1.29)	1.37 (0.89–2.10)
4+	36 (71.7)	93 (28.3)	0.62 (0.41–0.94)	0.93 (0.52–1.65)

^a^P-values are based on a Wald joint significance test

^b^Excludes Cesarean, suction and pre-term births

^c^Not shown in the table, the full model controlled for ethnicity, religion, occupation of household head, relationship to household head, age at first pregnancy, marital status, maternal occupation, wealth, source of trust for pregnancy-related questions, knowing a CHW, problems during ANC and number of ANC visits

^d^In each bivariate analysis: place of delivery (*n* = 907), education (*n* = 894), age (*n* = 906), parity (*n* = 904)

^e^When analyzed as a binary coefficient (none versus any), education is significant in the multiple regression (*p* = 0.01)

Women were more likely to stay 24 h if they delivered at a higher-level facility in all three types of analyses (including the single, full and final models). In the final model, only higher level of facility care continued to remain significantly associated with duration of stay.

In terms of education, in the bivariate logistic regression there appeared to be a dose–response between increasing education and delayed discharge, but there were not significant differences comparing women at the highest level of education with women possessing no education. Education was not a significant predictor of time to discharge in the full or final models and a dose response was not observed in point estimates.

Age was significant in bivariate logistic regression, significant in the full model but no longer significant in the final model (*p* = 0.07). In all models, it appeared that women younger than 19 are more likely to depart after 24 h compared to women older than 20. In terms of parity, the bivariate model showed that as women have more children, they are less likely to stay 24 h. However this finding was not statistically significant in the full or final models.

In the bivariate and full models, time to discharge did not differ significantly by ethnicity, religion, occupation of household head, partner education, marital status, knowing a community health worker, wealth, occupation, age at first pregnancy, problems during pregnancy and number of antenatal visits.

Being visited by a CHW during pregnancy was significant in bivariate analysis (*p* = 0.02), but could not be included in the full model due to a low response rate (*n* = 312).

In terms of receipt of postpartum care messaging, women staying 24 h or more at hospitals (60.4 %) and health care centers (56.6 %) appeared more likely than women who left sooner (54.8 % and 47.3 %, respectively) to receive at least one postpartum message on family planning, breastfeeding, or danger signs. However, after applying a Bonferroni correction, none of these differences were significant. In dispensaries, the trend in messaging was reversed among women who stayed longer than 24 h (29.3 %) versus less than 24 h (36 %), but again there were no statistically significant differences.

### Qualitative interviews: How a facility's structural limitations and routines compel an early departure

In 24 qualitative interviews with women who had delivered in a health center, 21 reported leaving the facilities within 24 h. The qualitative study was unable to capture an adequate number of women who discharged later than 24 h; of the 3 women who stayed longer than 24 h, two women delayed departure to take advantage of a health center’s vaccine day, and one was required to stay until her family paid outstanding fees. Women described their discharge time as primarily determined by providers. Women were content to leave before 24 h due to discomfort associated with maternity wards and a desire to return home. Women and their husbands frequently mentioned 24 h as a recommended time to stay, but this was viewed as necessary only in the event of a delivery complication. However, two women who reported experiencing “extra bleeding” during delivery said that they stayed for shorter periods (4 h and 20 h, respectively), and said their blood loss was not serious enough to merit a longer stay. No respondent highlighted issues related to observation of newborns as a factor that influenced time to discharge or duration of stay. For a breakdown of qualitative factors that compel an early discharge, see Table 3.

**Table 3:** The role of facilities in influencing time to discharge from interviews with women (*n* = 24), husbands (*n* = 15), and village leaders (*n* = 5)

1. Facility based limitations and routines
1) Pressure to discharge healthy mothers to accommodate others a) Limited supply of beds 2) Mass discharges regardless of delivery time a) “I left when the doctor was doing his rounds” 3) Discharges correspond with operating hours a) “I left when the dispensary was closing (for the day)”
2. Facility factors’ effect on those who accompany women
1) Facilities are uncomfortable for companions a) Facilities lack places to cook, sleep or do laundry i) Companions are left sleeping on floors, outside
3. Facility factors’ effect on women^a^
1) Physical Discomfort - “Hospitals are uncomfortable” a) Crowding and noise i) Lack of beds, women doubling up on beds ii) Constant noise from babies, fellow patients iii) No space b) Lack of cleanliness i) “There is dirtiness everywhere” ii) “The hospital’s mosquito nets stink” iii) “Hospitals are full of illness and disease” c) Wanting water or food i) No water, insufficient water, long queues to fetch water, no access to hot water (for a post-birth hot water massage) ii) No food nearby, food nearby is too costly, nowhere to prepare food, nobody to prepare food 2) Mental Discomfort- “I could not be at ease” a) Not enough attendants, adequate number of attendants but attendants are rude b) Women feel guilty “taking beds” from those “who really need it” c) Nearby patients are ill, unconscious or dying; their families are grieving

^a^To a lesser extent, women described other factors not related to facilities that compel departure: needing to care for children at home or leaving when transport was available

### Facility-based limitations and routines

The main reason for early discharge, reported by women in all districts as well as their husbands and local community leaders, involved personal expectations, provider encouragement to leave and an understanding that an early discharge frees space for others and is more comfortable for mother-baby pairs. The following quote illustrates pressure that women feel.

“Many pregnant mothers are waiting. When you leave, another woman gets the bed. Our wards are few and we are many. Like if you were to continue staying you would bring trouble for others… and the nurses tell us, ‘Now the time has come. You should go so that others can come.’”

- Woman in Ulanga District

Emphasizing bed limitations, mothers became forceful in describing a pressure to leave.

“Look! The space is not enough. And others are still there so you have to sleep two in one bed. One mother with a big stomach on this side and you with your baby on that side. It’s better you just go home.”

-Woman in Ulanga District

“Our hospital needs beds! Many people are just sleeping on the floors. Other people are sleeping two per bed. Some have had operations and others have delivered safely. Those with operations are given priority to lie in beds.”

- Woman in Kilosa District

At least 1 woman in each district described leaving once a doctor or other discharging authority made rounds or providers departed for the day. Women describe being discharged when rounds are conducted - regardless of time of delivery.

### Facilities as a source of unease for women and companions

Difficulties reported across districts related to facility infrastructure include an inability to obtain water to prepare foods as well as excessive noise. Mothers detailed how a lack of water inhibited cooking or laundering, bathing (to remove “the stink of birth”) and hot water massages (cleansing the lower back and groin area, which all women said they needed). Food was also a concern, namely a lack of food at facilities or cooks to prepare food (unless a mother had an able-bodied friend or family member to assist her). Mothers also noted that they feel guilty if those who accompany them feel uncomfortable- because they have no place to sleep or food to eat. One mother, who left on her husband’s bicycle 1.5 h after birth, said she would have liked to rest longer but the facility was under construction and the doctor ordered her to leave. Four women discussed noise at facilities in detail, and underscored how peaceful their homes felt compared to a facility.

“You stay there and that baby is crying, this one is crying … you cannot sleep. … I said if I have problems I will just return (to the facility) …. My home is home.”

– Woman in Kilosa

Less often, women described concerns about a lack of cleanliness, describing one facility as “a place with just no cleanliness at all”. Two mothers described a fear of contracting an illness at a facility with one woman describing how torn bed nets can lead to malaria and another woman from Kilosa describing how congestion and crowded rooms can cause disease: “where there is congestion (pause) there are diseases. Everyone carries her own diseases.”

One mother described sadness about facilities, which she associated with death and dying.

“I personally … just don’t like to be there. It makes me feel bad when I see a patient … unmoving or unconscious. To see people who are sick. It’s like that for me. If I’m safe, I go.”

- Woman in Ulanga

### Other factors that compel early departure

Three mothers mentioned a need to care for children or elderly family members at home. Four women described leaving when transportation (bicycle or bus) was available.

### Suggestions for promoting longer stays

Mothers most often suggested that providers should be educated on optimal stays after facility delivery, and that conditions and services in facilities should be improved. Less often, women suggested increasing the facility staff and encouraging compassion on the part of health professionals. On this point regarding respectful maternity care, community leaders were more forceful. A leader in Mvomero District said nurses berated women during delivery, which compelled them to leave early. A leader in Ulanga District emphasized the importance of medical authority and the need for providers themselves to be educated on the benefit of longer stays, “Once they tell a woman to stay, she will stay. The provider must learn first that she should stay. If the family knows it’s for the best interest of the mother, they’ll support it.” Medical authority proved very strong across respondent groups. When asked what would happen if a mother or her husband requested to stay longer (such as 24 h), mothers and husbands regarded this as an insult to the authority of medical professionals.

“Stay longer … if you have delivered safely you can’t stay. The only time they say you stay for 24 h is if you have problems. We know the rules.”

- Woman in Ulanga

“…it is the doctor who has that decision…He is the one who says when she can go. I don’t have the decision to say, ‘Wait.’”

- Husband in Morogoro Rural

## Discussion

### Main Findings

Within the first 24 h of facility delivery, 65.7 % of women with a normal, vaginal delivery interviewed as part of a cross-sectional quantitative survey reported being discharged. Among the study population, discharge within 24 h of facility delivery was described as a social norm and routine facility practice. Women expect to leave early unless they have undergone a cesarean section or experienced a complication, although even under these circumstances 10.5 % and 44.2 % of women left before 24 h, respectively. Our quantitative and qualitative findings indicate that the level of facility care and comforts existing or lacking in a facility have the greatest effect on time to discharge. This suggests that individual or interpersonal characteristics play a limited role in deciding whether a woman should stay for shorter or longer periods. Rather a woman is more likely to be discharged (or to feel disinclined to stay) if the facility does not support a longer stay. Our study has also highlighted that postpartum counseling – a critical feature of the immediate postpartum period – is limited in this setting regardless of time to discharge and that considerations related to newborn health are not at the forefront of a family’s mind when considering whether or when to leave. A discharge that occurs too soon represents a missed opportunity to assess the physical, psychological, and social wellbeing of the mother and newborn and to introduce and reinforce healthy practices during a highly vulnerable moment of life. Nevertheless, mothers expect an early discharge and can vividly describe how physical or emotional discomforts, coupled with provider or family-escort expectations compel an earlier departure.

### Interpretation

Fifty years ago, proponents of early discharge in high-income countries described shorter stays as a way to re-focus birth toward a family’s needs and away from a model considered hyper-technical, patriarchal and illness-based [22–25]. Under this model, an earlier return home fostered increased maternal confidence, family bonding and early sibling involvement, and served as an opportunity for mother-baby pairs to adapt to the rhythms of their family life rather than the routine of a hospital [25]. Literature from high-income countries has questioned the safety of early discharge particularly as it relates to increased maternal and infant readmissions [4, 22], breastfeeding problems and early breastfeeding cessation [26, 27], and a compromised ability to identify problems such as jaundice or sepsis in newborns [27–29], or depression, fear, stress, constipation or insomnia in mothers [27, 30, 31].

Our findings indicate that early discharge, as practiced in Tanzania, is routine and linked to family and provider expectations, which are guided by pragmatic limitations. Unlike studies in wealthy nations that uncovered social patterns in facility discharge with younger [24, 30], less educated [24, 32], or poorer [32, 33] women leaving earlier, this study found that early discharge is a norm which has little or no association with socio-demographic factors. Women described stays of longer than 24 h as necessary only for those who experienced major delivery complications or underwent surgery. The role of medical providers in deciding when a woman should depart was strong, which is reflected in other research on the power of medical authority [24, 34, 35]. The role of maternal education in determining time to discharge was somewhat ambiguous though generally positive. The importance of monitoring newborn health was not discussed by mothers as a factor that compelled a longer stay, which may be linked to limitations in facility infrastructure related to newborn health. According to the country’s most recent Service Provision Assessment, just 16 % of facilities offering delivery services have an emergency respiratory support system (an infant-sized ambu bag) for the newborn and only 3 % have an external heat source for newborns [17].

At least two studies in Tanzania have highlighted concerns of ill-health and social disharmony among women in the postpartum periods including: maternal depression, nervousness, fatigue, questions about infant health and infant crying, family or partner tensions, breastfeeding concerns and stress related to balancing one’s workload in light of new demands [36, 37]. Studies have also documented how Tanzanian mothers and fathers are eager for more medical guidance as it relates to maternal and newborn health, particularly concerning timing of sexual resumption, safety and appropriateness of contraception and conflicting messages they receive on this topic [37, 38]. We found that postpartum messaging was limited, across all facility types, but especially in dispensaries. We also found no evidence that staying longer than 24 h increased the probability that women would receive postpartum care messages regarding breastfeeding, maternal or newborn danger signs, or family planning.

We present two options to address the situation of early discharge. A first proposed course of action would be to view early discharge as highly problematic and worthy of significant investment. This approach would entail policy-level reiterations of a minimum required number of hours of stay, clarification of these policies to health providers and, most importantly, massive investments in facility resources and infrastructure including increases in human resources, beds and access to food and running water (including heated water) and/or the mobilization of skilled personnel who can follow-up with women and their newborns in communities during the early postpartum period. Following these investments, behavior change communication campaigns to promote longer postpartum stays and encourage family uptake of longer stays could be undertaken.

A second proposed course of action would view the current situation as sub-optimal, but would stop short of broad changes in favor of a targeted focus on most-critical concerns. In this case, the status quo for women with normal deliveries would be maintained, but all women who have experienced a complication, given birth via cesarean section or given birth to a preterm or low birth-weight baby would be supported in postpartum stays of at least 24 h.

Under either course of action, we strongly support WHO and UNICEF joint statements promoting early postnatal home visits as a complementary strategy to improve coverage of care and newborn survival [12]. We also encourage investments in pre-discharge, postpartum counseling on breastfeeding, danger signs and family planning, including investments in personnel education and counseling promotion at all facility levels.

Until more research is conducted in this and similar low-income settings, we view a targeted, selective approach as necessary. However, we caution against enforcement of policies on a minimum number of hours that all women must stay in the absence of improvements to infrastructure, and provider deployment and training on respectful, non-discriminatory, culturally competent care [39]. We state this because an emerging body of research has highlighted dimensions of disrespect and abuse experienced in the hours surrounding birth, including detention of mothers in facilities after childbirth [40–46]. For this reason, while we urge that departure after birth should be delayed especially in instance of complicated deliveries, the creation and enforcement of policies on minimum stays must be undertaken in an environment that respects women's autonomy and meets internationally-recognized guidelines for mother- baby friendly birthing facilities [39].

### Strengths and limitations

This study is strengthened by the fact that it drew upon both a survey and in-depth interviews. Due to the cross-sectional design of the quantitative survey, it demonstrates association without the ability to attribute direction or causality. The study is limited in terms of recall bias as respondents may have difficulty remembering details in the postpartum period, or from an event that may have occurred several months ago. It would have also been helpful to capture provider perspectives on early discharge in order to understand the clinical knowledge and decision-making processes that guide times to discharge. Due to an inability to capture an adequate number of women who discharged late, this study did not reach saturation on qualitative findings related to reasons for discharging after 24 h. Finally, as the qualitative interviews focused on problems identified as most important by women, the specific issue of postpartum stay was not explored in equal depth across all interviews.

## Conclusions

In the short-term, findings from this study call for targeted emphasis on the need for longer stays among women who have experienced complications during delivery or cesarean section or have given birth to a pre-term or low birth-weight baby. In the longer term, investments in human resources, infrastructure and critical supplies – such as beds and water – coupled with education among providers and mother-escort pairs on the importance of 24-h stays could foster delayed facility discharge and improve maternal and child health. Implementation and enforcement of any policy promoting longer stay must consider women’s and providers’ existing perceptions and experience of early discharge as a desirable practice.

## Additional files

Additional file 1:
**Quantitative Tool (Excerpts).** (PDF 197 kb) Additional file 2:
**Qualitative Tool.** (PDF 117 kb)
