# Supplementary material for: “You should go so that others can come”; the role of facilities in determining an early departure after childbirth in Morogoro Region, Tanzania
Source: BMC Pregnancy Childbirth. 2015 Dec 9;15:328. doi: 10.1186/s12884-015-0763-1 (PMC4675015; doi:10.1186/s12884-015-0763-1)
Supplement: Additional file 1: — Quantitative Tool (Excerpts). (PDF 197 kb) [file 12884_2015_763_MOESM1_ESM.pdf]

104. Comments

2. Interviewee's personal information.

201.	When were you born? Please tell me the date, month and the year you were born	------------/---------/--------------	
202.	What is your age, in terms of years?<br><br>***COMPARE AND THEN CORRECT 201 AND/OR 202 TO ENSURE CONSISTENCY.***	Write down the age, in terms of years	[ ]
203.	What is your religion?	1. Christian<br>2. Muslim<br>3. Hindu<br>4. Budha<br>5. Others (Mention) _______________	[ ]
204.	What is your tribe?	1. Luguru<br>2. Kaguru<br>3. Pogoro<br>4. Ndamba<br>5. Others (Mention) ______________	[ ]
205.	What activity are you engaged in to earn living?	**Agriculture**<br>1. Crops / Animal keeping<br>2. Fishing<br>**Employed**<br>3.Government and other institutions<br>4.Private sector<br>**Self employed (Not in agriculture/Animal keeping)**<br>5. Has employed other people<br>6. Has not employed anyone<br>7. S/he is involved in family projects (non-agricultural projects) without gaining any payment(s).<br>**Not working**<br>8. S/he is ready to work<br>9. S/he is not ready to work<br>10.Dealing with domestic activities/housewife<br>11. Student	[ ]

		12. Can not work (Old age, retired, sick, disabled)<br>13.Other activities<br>(Mention) ___________________________	
206.	What is your relationship with the head of this household?	1. Head of household<br>2. Wife<br>3. Daughter<br>4. In-law (Married)<br>5. Grand daughter<br>6. Mother<br>7. Mother in-law<br>8. Sister<br>9. Sister in law<br>10. Relative<br>11. Adopted/step daughter<br>12. Domestic servant<br>13. Friend<br>14. Others-not relatives based: (Mention) ________________	[ ]
207.	What is the head of household doing to earn the living? (income generating activity done by the head of household)	**Agriculture**<br>1. Crops / Animal keeping<br>2. Fishing<br>**Employed**<br>3.Government and other parastatals<br>4.Private sector<br>**Self employed (Not in agriculture/Animal keeping)**<br>5. Has employed other people<br>6. Has not employed anyone<br>7. S/he is involved in family projects (non-agricultural projects) without gaining any payment(s).<br>**Not working**<br>8. S/he is ready to work<br>9. S/he is not ready to work<br>10.Dealing with domestic activities/housewife<br>11. Student<br>12. Can not work (Old age, retired, sick, disabled)<br>13.Other activities<br>(Mention) __	[ ]
208.	Have you ever been to school for formal education?	1. Yes<br>2. No ***(GO TO QUESTION 210)***	[ ]
209.	What is your level of eduvation?<br>That is ,how many years did you spend in school or college/university?	Write down the number of years spent in attending school or college/university.	[ ]
210.	Now, i request you to read the following sentence.<br><br>***SHOW THE CARD WITH THAT PARTICULAR SENTENCE TO THAT PARTICIPANT. IF THE PARTICIPANT CAN NOT READ THE WHOLE SENTENCE,***	1. Was able to read the whole sentence<br>2. Was able to read some parts of that sentence<br>3. can not read the given sentence<br>4. Blind/has eye(s) problems	[ ]

	INVESTIGATE: Can you read any part or any words from this sentence?		
211.	Are you currently married or do you live with any man in any marital relationship?	***1. Yes (GO TO QUESTION 213)***<br>2. No	[ ]
212.	**If NO,** what type of marital relationship are you currently in? Widow, divorced, separated from your partner?	1. Widow<br>2. Divorced<br>3. Separated from my partner<br>**4. Never been married – GO TO QUESTION 215**	[ ]
213.	Has your husband or your partner got formal school education?	1. Yes<br>2. No<br>9. I don't know/I don't remember	[ ]
214.	What is the education level of your husband or partner?	Mention the years spent in school	[ ]
215.	How old were you when you became pregnant for the first time?	Years ____	[ ]
**Please let me ask you few questions concerning your household.**			
216.	What is the main source of drinking water and water for other uses in your household?	1. Tap water,, available inside the house<br>2. Tap water, available outside the house<br>3. Tap water, from the community water supply.<br>4. Open water well, found outside the house<br>5. Open well for community uses<br>7. Constructed water well, located outside the house<br>8.Springs/Rivers/water channels/pond/lake<br>9. Other (Mention)___________________	[ ]
217.	Is your household with the toilet?	1.Yes<br>2.No ***(GO TO QUESTON 219)***	[ ]
218.	If yes, what type of toilet is it?	1. Toilet not using water to flash waste materials (not shared one).<br>2. Toilet using water to flash waste materials (shared with other household(s))<br>3. Pit latrine,allowing air in but not shared with other household(s).<br>4. Pit latrine, allowing air in and shared with other households.<br>5. Toilet constructed under traditional settings but not shared with other households<br>6. Toilet constructed under traditional settings and shared with other households<br>7. other type(s) (Mention)________________	[ ]

219.	What are the materials used to roof the house where the head of this household lives in?<br><br>**(INVESTIGATE AND KEEP THE RECORD OF THINGS YOU WILL SEE)**	1. Grasses/ Leaves/ Soil<br>2. Roofing iron sheets<br>3. Tiles<br>4. Cement<br>5. Asbestos<br>6. Another type (Mention)________________________	[ ]
220.	What is are the materials used to make the walls of that house?<br><br>***(KEEP THE RECORD OF THE THINGS YOU WILL SEE. IN CASE MORE THAN ONE MATERIAL HAS BEEN USED, SHOW THAT ONE THAT HAS BEEN USED MAINLY)***	1. Trees and soil<br>2. building blocks made using soil<br>3. building blocks burnt in furnaces<br>4. Timber<br>5. Cement blocks<br>6. Stones<br>7. other materials (mention) __________	[ ]
221.	What are materials used in making the floor of that house?<br>**(INVESTIGATE AND KEEP THE RECORD OF THE THINGS YOU WILL SEE)**	1. Soil/Sand<br>2. Animal secretions<br>3. Wood planks/bamboo trees<br>4. Polished timber<br>5. Ceramic<br>6. floor tiles<br>7. Cement<br>8. Other (Mention) _____________	[ ]
222.	Is the house mentioned above owned by your household?	1.Yes ***(GO TO QUESTION 224)***<br>2. No	[ ]
223.	If this household doesn't own this house, have you rented it?	1.we have rented the house<br>2. another arrangement (Mention) ___________	[ ]
224.	Does your household own land?	1.Yes<br>2. No ***(GO TO QESTION 226)***	[ ]
225.	If your household owns the land, what is the size in acres ?	Write down(an estimation) the number of acres<br>998 I don't know	[ ]
226.	Is your house with electric power?	1.Yes<br>2.No	[ ]

227.	Is your house with the following?(put a mark on the corresponding item)<br>A. Radio and music instruments<br>B. chickens or ducks<br>C. Cow or donkey<br>D. Sheep, goat, pig<br>E. Bicycle<br>F. motorcycle<br>G. car or tractor<br>H. Television<br>I. Fridge<br>J. mobile phone<br>K. Home telephone (not mobile phone)<br>L. shop/kiosk/bar<br>M. sawing machine<br>N. Sofa/Sofa set.<br>O. Special table for food.	A. 1=YES 2=NO<br>B. 1= YES 2=NO<br>C. 1= YES 2=NO<br>D. 1= YES 2=NO<br>E. 1= YES 2=NO<br>F. 1= YES 2=NO<br>G. 1= YES 2=NO<br>H. 1= YES 2=NO<br>I. 1= YES 2=NO<br>J. 1= YES 2=NO<br>K. 1= YES 2=NO<br>L. 1=YES 2=NO<br>M. 1=YES 2=NO<br>N. 1=YES 2=NO<br>O.1=YES 2=NO	[ ]<br>[ ]<br>[ ]<br>[ ]<br>[ ]<br>[ ]<br>[ ]<br>[ ]<br>[ ]<br>[ ]<br>[ ]<br>[ ]<br>[ ]<br>[ ]<br>[ ]

3 Pregancy history and services received

May i please ask you about your history of prenancy and health services?

301.	Is there any biological child of yours that you are living with currently?	1.Yes<br>2.No	[ ]
302.	How many sons of yours are you currently living with?<br>How many daughters of yours are you currently living with?	***IF NO CHILDREN WRITE '00'***	[ ]<br>[ ]
303.	Do you have any biological children that you are not living with at this moment?	1.Yes<br>2. No **(Go to 305)**	[ ]
304.	If yes, how many sons that you are not living with currently?<br>How many daughters that you are not living with at this time?	The number of male children living far<br>The number of female children living far.<br>***IF NO CHILDREN LIVING FAR AWAY, WRITE '00'***	[ ]<br>[ ]<br>[ ]
305.	***REFER QUESTIONS NUMBER: (302 + 304), COMPARE AND THEN CORRECT TO CONFIRM THE REAL TOTAL NUMBER OF CHILDREN***<br>Currently you have________living children.	1.yes<br>2 .No	[ ]
306.	Have you ever given birth to a living child who died later?<br>***IF THE ANSWER IS NO PROBE: ANY***	1. Yes<br>2.No (**Go to question 308**)	[ ]

		E. Total costs__________________	

4 Health problems during delivery/intra-utero fetal death(s)

Now i request to ask you about the problems you faced during pregnancies

401	Where was the delivery of your baby conducted?<br><br>***IF SHE DOES NOT KNOW WEATHER IT WAS THE HOSPITAL, HEALTH CENTRE, OR GOVERNMENT CLINIC OR PRIVATE ONE: WRITE DOWN THE NAME OF WHERE SHE IS.***	1. Morogoro regiona hospital **(GO TO 403)**<br>2. Other hospitals (Mention)_____________________ **(GO TO 403)**<br>3. Health center (Mention)_________ **(GO TO 403)**<br>4. Dispensary (mention)_____________ **(GO TO 403)**<br>5. Pharmacy **(NENDA 403)**<br>6. Anther centre for health services (Mention)______ **(GO TO 403)**<br>7. At the home of the traditional birth attendant<br>8. At my home?<br>9. At the home of the community health worker<br>10. At the home of somebody else<br>11. Along the way	[ ]
402	If you did not deliver at the health centre, what was the reason(s)?<br>**1= she has mentioned; 2=she has not mentioned**<br><br>***(don't read out the answers but probe)***	A. A distance and high cost for transport<br>B. time for services does not correspond with schudule<br>C. Unpleasant behavior shown by health provider<br>D. Inappropriate attitude of the health care provider<br>E. Lack of confidentiality<br>F. Lack of medications<br>G. Long waiting time<br>H. High costs of the required service(s)<br>I. Religious reason(s)i<br>J. There is no any benefit(s)<br>K. I did not know the importance of doing so<br>L. I did not the permission to do so<br>N. any other reasons (mention)	[ ]<br>[ ]<br>[ ]<br>[ ]<br>[ ]<br>[ ]<br>[ ]<br>[ ]<br>[ ]<br>[ ]<br>[ ]<br>[ ]<br><br>[ ]<br>[ ]

403	Who helped you at the time of delivery?<br><br>***(DO NOT READ OUT THE ANSWERS BUT PROBE OR ASK: OTHER REASON(S)? 1= SHE MENTIONED 2= SHE DID NOT MENTION)***	A. Heath worker at the dispensary<br>B. Health worker at the health center<br>C. Health worker in the hospital<br>D. Health worker in the village<br>E. Traditional healer<br>F. Traditional birth attendant<br>G. Brother/sister/friend/neighbour<br>H. Another one<br>I. She doesn't know/she doesn't remember	[ ]<br>[ ]<br>[ ]<br>[ ]<br>[ ]<br>[ ]<br>[ ]<br>[ ]<br>[ ]
404	Did the village health worker advice that you go to deliver at the health center?	1. Yes<br>2. No	[ ]
405	For how many hours did your labour pain last?<br>(that is, how long did pain associated with labour last?<br><br>**PROBE**	minutes_________________<br>hours____________________<br>days ___________________<br>***(write the total number of minutes)***<br>I don't know= 00	[ ]
406	Did you get any problem during delivery or immediately after delivery?	1= yes ,<br>2= No (**GO TO 409**)	[ ]
407	If yes what at those problems did you face during delivery or shortly after delivery?<br><br>**DANGER SIGNS ARE ON THE RIGHT SIDE. GIVE THE RESPONDENT A CHANCE TO CHOOSE FROM EVERY ANSWER PUT FORWARD BY SAYING:**<br><br>**1=YES, 2=NO**	2 Too much bleeding through the vagina<br>3 eclampsia<br>4 Swelling of the legs and the face?<br>5 Difficult in baby's descent(i.e. baby moving through the birth canal with difficult)?<br>6 Placenta coming out with difficulty<br>7 Loss of consciousness<br>8 Other signs_________________________	[ ]<br>[ ]<br>[ ]<br>[ ]<br>[ ]<br>[ ]<br>[ ]
408	Did the village health worker advice you to seek treatment for that problem from the health centre?	1. Yes<br>2. No	[ ]
409	What was your method of delivery?	1=normal method<br>2= operation(caesarean section)<br>3= by suction using iron materials<br>4= another method (mention)……………<br><br>**IF SHE DELIVERED WHILE AT HOME, GO TO NUMBER 422.**	[ ]

410	Before discharge, after (NAME) was born, was there any health worker who investigated and/or checked into your health?	1=Yes<br>2=No (**GO TO 412**)	[ ]
411	If yes,<br><br>***READ OUT ANSWERS FOR HER***<br><br>***WRITE***<br>***1= YES 2= NO)***	A. Did the health worker emphasize that you stay with the baby till when you were discharged from the heath centre? (Yes/No)<br>B. Did the health worker emphasize that you eat and drink regularly? (Yes/No)<br>C. Did the health worker instruct you to press your uterus on regular bases? (Yes/No)<br>D. Did the health worker check on you for danger signs on regular bases?(Yes/No)<br>E. Did the health worker insist that you go to the toilet for a short call often? (Yes/No)<br>F. Did the health expert help you during the initiation of breastfeeding of the baby? (Yes/No)	[ ]<br>[ ]<br>[ ]<br>[ ]<br>[ ]<br>[ ]
412	How much time did it take for you to be discharged from the health center after the delivery?	minutes ________________<br>hours___________________<br>days ___________________<br>***(write down the total number of minutes)***<br>I don't know= 00	[ ]
413	Were you given any professional advice before discharge from that centre/place of delivery?	1=yes<br>2= no (**GO TO 424**)<br>9= she doesn't know	[ ]

414	Before discharge, were you advised to breastfeed the baby continuously without giving him/her any other foods?<br><br>[continuous breastfeeding means that the baby is living only on mother's breast milk and no any other thing such as water, porridge or any other thing for the period of six months].	1=yes<br>2= no	[ ]
415	What are the benefits of continuous breastfeeding the the child without any other foods for the period of first six months?<br>1. She mentioned<br>2. She did not mention	A. it is one of the methods of preventing pregnancies because during this period normally you have not gained back menses<br>B. to prevent transmission of HIV from the mother to the baby<br>C. it is the nice food/diet the young baby	[ ]<br>[ ]<br>[ ]
416	Were you advised to attend the children clinic at that center?	1=Yes<br>2= No **(GO TO 418)**	[ ]
417	When were you told to attend the children clinic?<br><br>**READ OUT ALL ANSWERS, WRITE 1= YES, 2=NO**	A. Within 24 hours<br>B. Within 7 days after delivery<br>C. On the 28th day after delivery<br>D. on the 42nd day after delivery<br>E. six months after delivery	[ ]<br>[ ]<br>[ ]<br>[ ]<br>[ ]
418	Were you advised to get important supplements for your health?	1=Yes<br>2= No	[ ]
419	Were you advised on getting medication for boosting the amount of blood in your body	1=yes<br>2= no	[ ]
420	Were you told anything concerning family planning and the use of family planning methods?	1=yes<br>2= no **(GO TO. 422)**	[ ]
421	Please, let me now ask you about what you were told concerning family planning after delivery<br><br>**READ OUT ALL ANSWERS FOR HER.**<br>**WRITE 1= YES, 2=NO**	A. Were you told anything concerning family planning?<br>B. Were you told when you could regain your ability to once again become pregnant after delivery or termination of pregnancy?.<br>C. Where you told to go and get famly planning medications from the healh center?<br>D. Were you told how to involve your husband/partner in the issues of family planning?<br>E. Were you told that if you continued to breastfeed, you will have an extended period of ammenorrhea?<br>F. Were you given any other advice on family planning? Please mention _______________	[ ]<br>[ ]<br>[ ]<br>[ ]<br>[ ]<br>[ ]<br>[ ]
422	Were you told how to identify the danger signs on post delivery women?	1=Yes<br>2= No	[ ]

423	Were you advised on how to identify the danger signs that happens to young babies?	1=Yes<br>2= No	[ ]
424	Did you pay any money to get health services related to delivery or to get treatment in relation to your health problems during delivery?	1= Yes<br>2= No **(GO TO 426)**	[ ]<br>[ ]
425	If yes, how much did you pay for the following services?<br><br>***PROBE FOR EACH SECTION AND WRITE DOWN THAT AMOUNT IN SHILLINGS.***<br><br>***WRITE '0' IF NO MONEY WAS SPENT***<br>***WRITE '9' IF SHE DOES NOT KNOW***	A. Medications an vitamins__________________<br><br>B. Counselling costs ___________________<br><br>C. transport ___________________<br><br>D. .other costs (mention) ________________<br><br>**IF THE RESPONDENT KNOWS THE TOTAL COSTS, WRITE DOWN THAT FIGURE HEREUNDER,**<br><br>E. Total costs___________________	[ ]

After delivery; please let me now ask you about the services after delivery.			
426	Did the village health worker visit you after delivery?	1=Yes<br>2= No *(GO TO 432)*	[ ]
427	How many times did he/she visit you?	***WROTE DOWN THE NUMBER OF TIME OF THOSE VISITS***	[ ]
428	When she visited you, was it within 24 hours after delivery at home/after discharge from the heath center?	1=Yes<br>2= No	[ ]
429	Did s/he visit you in any of following moments listed here?<br><br>**READ OUT ALL ANSWERS FOR HER. WRITE 1= YES, 2=NO**	A. Within one week after delivery<br>B. Within 2-4 weeks after delivery<br>C. On the 42nd day after delivery<br>D. Six months after delivery	[ ]<br>[ ]<br>[ ]<br>[ ]
