# Supplementary material for: “You should go so that others can come”; the role of facilities in determining an early departure after childbirth in Morogoro Region, Tanzania
Source: BMC Pregnancy Childbirth. 2015 Dec 9;15:328. doi: 10.1186/s12884-015-0763-1 (PMC4675015; doi:10.1186/s12884-015-0763-1)
Supplement: Additional file 2: — Qualitative Tool. (PDF 117 kb) [file 12884_2015_763_MOESM2_ESM.pdf]

Qualitative Instruments

Investigator's note: Qualitative research is an iterative process. As more is learned about a topic, additional questions are formulated and asked to gain a deeper understanding of the topic. The questions in this guide represent our starting point for the interviews in this phase of the Morogoro Evaluation Project. Over the course of the data collection, the questions evolved. More probes were added for some questions.

Interviews with women

The Morogoro Evaluation Project<br>Muhimbili University of Health and Allied Sciences (MUHAS) | Johns Hopkins School of Public Health<br>Integrated Maternal and Newborn Health Care Program in Morogoro Region, Tanzania

Location of household where user lives

101. RESPONDENT ID CODE	
102. District/Wilaya	
103. Town or village/Mji au Kijiji	
104. Ward/kata	
105. Ten cell leader/Balozi	
106. Head of household (male or female)	
107. Nearest Health Centre/Kituo cha Afya	
108. Nearest Dispensary/Zahanati	

Introductory Script

"Good morning/afternoon, I would like to thank you for giving the time to speak with us in this interview in which we would like to learn more from you about maternal, newborn, and child health care in Tanzania. Firstly, let me introduce myself. I am [name]. I am with a team of researchers engaged by Muhimbili University of Health and Applied Sciences (MUHAS) to conduct a study in collaboration with the Ministry of Health on maternal and child health in Morogoro. Our main focus is to hear your opinions about maternal and child health services in this area.

In the discussion we will be interested to hear your opinions. As such there are no right or wrong answers because you are only expected to share your experiences. Your name will not be reported as a participant in the study.

Because this is a research study approved by the ethical review board at MUHAS, we need to ask for your informed consent before proceeding. I will read through the oral consent form now . . .

[Read out informed oral consent]

Is it okay to proceed?"

User Demographic Information

201. Respondent ID#:
202. District:
203. Age:
204. Marital Status:
205. What language do you speak at home?
206. What is the highest level that you have studied?
207. How well do you read and write Swahili?
208. What other languages can you read and write?

Introduction to the Interview

"I would now like to speak with you about your experiences seeking health care services in Morogoro. Specifically, I would like to talk about your impressions about the current health services focusing on the health of pregnant women, new mothers and newborns and factors that influenced why and from where you sought care"

Pregnancy and Delivery care-seeking

301. During your **last pregnancy** did you seek medical care for any reason – preventative or curative?
    a. Probe
        i. If yes, can you please tell me more about why. If no, can you tell me more about why not.
        ii. From where did you seek care?
        iii. From whom did you seek care and why?
        iv. Can you tell me more about the factors that influence why, when and from whom you choose to seek care?
        v. Who in your family makes the ultimate decision on matters such as this?

302. From the moment you knew (name of baby) was coming, please tell me the story of your delivery.
    a. Probes:
        i. Where did delivery occur?
        ii. How were decisions made about where to deliver?
        iii. Who in attendance during delivery?
        iv. How felt during delivery?
        v. How newborn and mother cared for immediately after delivery?
        vi. Experiences immediately postpartum
            1. When depart and how decide when depart?
            2. Nature of information received (if any) prior to departure

User experience with health system

401. Can you tell me more about your perceptions of the maternal, newborn and child health services provided in government health facilities?

402. Can you tell me more about your perceptions of the maternal, newborn and child health services available at the community level?

403. How far away is the nearest government health facility from your home (round trip time in minutes/ hours)?
    Probes
        i. How would you travel to this health facility?
        ii. What are the average expenses you would likely incur?

404. Are there any critical services that you feel to be required but which are presently not available? If yes, please describe.

Support from family and community for MNCH care seeking

We have talked about a lot of things, about your careseeking during labor and delivery especially. Now I would like to ask you about the support that you receive from your family members and community.

501. What support was provided to you by your family during

a. Your pregnancy
b. During you delivery
c. To you during the first 42 days post-delivery
d. To your newborn baby with the first 28 days of life.

502. What are the barriers you face to seeking preventative care for MNCH?

503. What are the barriers you face to seeking curative care for MNCH?

Interviews with husbands of women

The Morogoro Evaluation Project<br>Muhimbili University of Health and Allied Sciences (MUHAS) | Johns Hopkins School of Public Health<br>Integrated Maternal and Newborn Health Care Program in Morogoro Region, Tanzania

Location of household where user lives

109. District/Wilaya	
110. Town or village/Mji au Kijiji	
111. Ward/kata	
112. Ten cell leader/Balozi	
113. Head of household (male or female)	
114. Nearest Health Centre/Kituo cha Afya	
115. Nearest Dispensary/Zahanati	

Introductory Script

"Good morning/afternoon, I would like to thank you for giving your time to speak with us in this interview in which we would like to learn more from you about the health of mothers and children in Tanzania. Firstly, let me introduce myself. I am [name]. I am with a team of researchers engaged by Muhimbili University of Health and Applied Sciences (MUHAS) to conduct a study in collaboration with the Ministry of Health on maternal and child health in Morogoro. Our main focus is to hear your opinions about maternal and child health services in this area.

In the discussion we will be interested to hear your opinions. As such there are no right or wrong answers because you are only expected to share your experiences. Your name will not be reported as a participant in the study.

Because this is a research study approved by the ethical review board at MUHAS, we need to ask for your informed consent before proceeding. I will read through the oral consent form now . . .

[Read out informed oral consent]

Is it okay to proceed?"

User Demographic Information

209. Respondent ID#:
210. District:
211. Age:
212. Marital Status:
213. What language do you speak at home?
214. What is the highest level that you have studied?
215. How well do you read and write Swahili?
216. What other languages can you read and write?

Introduction to the Interview

"I would now like to speak with you about your thoughts on your wife seeking or not seeking health care services in Morogoro. Specifically, I would like to talk about your impressions about the importance or unimportance of seeking health care services for women who are pregnant, new mothers and newborns."

Pregnancy and Delivery care-seeking

301. During your wife's **last pregnancy** did she seek medical care for any reason – preventative or curative?
    a. Probe 1: If yes, can you please tell me more about it. Do you know why she sought care? If no, can you tell me more about why not.

b. Probe 2: From where did she seek care?
c. Probe 3: From whom did she seek care and why?
d. Probe 4: Did you accompany her? Can you tell me more about her experience seeking care from (person from whom she sought care)?
e. Probe 5: Can you tell me more about the factors that influence why, when and from whom your wife chooses to seek care?
    i. Probe: Who in your family makes the ultimate decision on seeking care? Why this person? How is a decision made?

302. Where did your wife's last delivery occur?
303. Can you please tell me more about the people who attended your wife's last delivery?
    a. Probe 1: Was a trained / skilled provider in attendance? If yes, can you please tell me more about why? If no, can you tell me more about why not?
    b. Probe 2: What factors influenced your wife's decision to deliver in the place you mentioned?
    c. Probe 3: How do you view the difference between have a skilled provider in attendance versus not having a skilled provider in attendance?
    d. Probe: Please tell me more about the time after the delivery until the time you and your wife departed the facility to return home.

User experience with health system

401. Can you tell me more about your perceptions of the maternal, newborn and child health services provided in government health facilities?
402. Can you tell me more about your perceptions of the maternal, newborn and child health services available within the community?
403. How far away is the nearest government health facility from your home (round trip time in minutes/ hours)?
    Probe 1: How would your wife travel to this health facility?
    - Probe: Husband involvement

    Probe 2: What are the average expenses your wife would likely incur?
404. Are there any private sector services available which your wife utilizes? If yes, which?
405. Are there any critical services which you feel to be required but which are presently not available to your wife? If yes, please describe.

Support from family and community for MNCH careseeking

We have talked about a lot of things, about child care, health care and careseeking. Now I would like to ask you about your opinions of health care and facility-based care seeking for your wife.

501. How do you feel about your wife going to health facility? Can you think of an example when your wife wanted to seek care, but you did not think it was necessary? Please tell me about this.
502. Can you think of an example when you wanted your wife to seek care in a facility but she did not want to? Please tell me about that.
503. How do your opinions today on formal health care compare with your opinions a few years ago?
504. How do your opinions on health care compare with the opinions of others in your community?
505. What are the barriers that you, as a family, face to seeking preventative care for your wife or child?
506. What are the barriers you, as a family, face to seeking curative care for your wife or child?

Interviews with religious and community leaders

Muhimbili University of Health and Allied Sciences (MUHAS)<br>Johns Hopkins Bloomberg School of Public Health<br>Evaluation of Integrated Maternal and Newborn Health Care Program in Morogoro Region, Tanzania

Introductory Script:

Good morning/afternoon, I would like to thank you for giving the time to speak with us in this interview in which we would like to learn more from you about maternal, newborn, and child health care in Tanzania. Firstly, let me introduce myself. I am [name] and I am with a team of researchers engaged by Muhimbili University of Health and Applied Sciences (MUHAS) to conduct a study in collaboration with the Ministry of Health on maternal and child health in Morogoro. Our main focus is to hear your opinions about maternal and child health services in this area.

In the discussion we will be interested to hear your and opinions. As such there are no right or wrong answers because you are only expected to share your experiences. Your name will not be reported as a participant in the study.

Because this is a research study approved by the ethical review board at MUHAS, we need to ask for your informed consent before proceeding. I will read through the oral consent form now . . .

[Read out informed oral consent]

Is it okay to proceed?

Guiding Questions

1. Can you tell me about your role in the community?

2. How do members of this community access health care?
    a. Is there anything that helps people access health care in this community compared to other communities?
    b. What are the difficulties in accessing health care?

3. What do you think are the main health problems in this community?

4. What health care services do pregnant women in this community use? How are they accessed?

5. Do pregnant women usually see a doctor during pregnancy?
    a. Who provides antenatal care and where is it provided?
    b. If women do not receive antenatal care, what are some of the reasons?
    c. For women that do receive antenatal care, what are some of the reasons?
    d. For women that do not receive antenatal care, is there anything that would make it easier or more appealing for them to attend antenatal care?

6. Where do women deliver their babies in this community?
    a. Who assists the women during delivery?
    b. For women that don't go to the facility for delivery, what are the reasons?
    c. For women that do go to the facility, what are the reasons?
    d. For women that don't deliver at the facility, is there anything that would make it easier or more appealing for them to deliver at the facility?

7. After a baby is delivered, does a health worker usually check the baby?
    a. Who usually checks the baby?

b. What do they do when they check the baby?
c. For women who don’t take the baby to the health center, what are the reasons they don’t go to the health center?
d. For women who don’t take the baby to the health center, is there anything that would make it easier or more appealing for them to take the baby to the health center?

8. In summary, what do you think are the biggest challenges for health care for pregnant women and new babies in this community? How can these challenges be solved?
